# Interventional cardiovascular magnetic resonance: state-of-the-art

**DOI:** 10.1186/s12968-023-00956-7

**Published:** 2023-08-14

**Authors:** Toby Rogers, Adrienne E. Campbell-Washburn, Rajiv Ramasawmy, D. Korel Yildirim, Christopher G. Bruce, Laurie P. Grant, Annette M. Stine, Aravindan Kolandaivelu, Daniel A. Herzka, Kanishka Ratnayaka, Robert J. Lederman

**Affiliations:** 1 Cardiovascular Branch, Division of Intramural Research, National Heart, Lung, and Blood Institute, National Institutes of Health, Building 10/Room 2C713, 9000 Rockville Pike, Bethesda, MD 20892-1538 USA; 2 Section of Interventional Cardiology, MedStar Washington Hospital Center, 110 Irving St NW, Suite 4B01, Washington, DC 20011 USA; 3 Johns Hopkins Hospital, Baltimore, MD USA; 4 Rady Children’s Hospital, San Diego, CA USA

**Keywords:** Interventional cardiovascular magnetic resonance, iCMR, Magnetic resonance imaging, Invasive cardiovascular magnetic resonance, Cardiac catheterization, Electrophysiology

## Abstract

Transcatheter cardiovascular interventions increasingly rely on advanced imaging. X-ray fluoroscopy provides excellent visualization of catheters and devices, but poor visualization of anatomy. In contrast, magnetic resonance imaging (MRI) provides excellent visualization of anatomy and can generate real-time imaging with frame rates similar to X-ray fluoroscopy. Realization of MRI as a primary imaging modality for cardiovascular interventions has been slow, largely because existing guidewires, catheters and other devices create imaging artifacts and can heat dangerously. Nonetheless, numerous clinical centers have started interventional cardiovascular magnetic resonance (iCMR) programs for invasive hemodynamic studies or electrophysiology procedures to leverage the clear advantages of MRI tissue characterization, to quantify cardiac chamber function and flow, and to avoid ionizing radiation exposure. Clinical implementation of more complex cardiovascular interventions has been challenging because catheters and other tools require re-engineering for safety and conspicuity in the iCMR environment. However, recent innovations in scanner and interventional device technology, in particular availability of high performance low-field MRI scanners could be the inflection point, enabling a new generation of iCMR procedures. In this review we review these technical considerations, summarize contemporary clinical iCMR experience, and consider potential future applications.

## Background

Cardiovascular magnetic resonance (CMR) imaging is considered by most a non-invasive diagnostic modality, wherein images are acquired over long breath-holds and reviewed retrospectively. In fact, it is possible to generate CMR images in real-time with frame rates approaching those of X-ray fluoroscopy. This has the potential to enable invasive cardiovascular procedures to be performed inside the magnetic resonance imaging (MRI) scanner. A fair critique of the field of interventional CMR (iCMR) over the last decade is that reality has lagged behind the promise. It is true that translation of novel pre-clinical applications has been frustratingly slow, largely because of the technical challenge of re-engineering catheter devices for use in the MRI environment. Nonetheless, diagnostic CMR catheterization procedures are a clinical reality at numerous institutions around the world today. Looking to the future, MRI hardware and software advances are enabling increasingly complex procedures to exploit the unique capabilities of CMR to image and to measure flow through individual blood vessels. In this state-of-the-art review, we summarize where the field of iCMR is today, and highlight how technological advances from the last decade will shape future applications.

## What iCMR procedures can we do today?

### Diagnostic cardiac catheterization

**Fig. 1:**
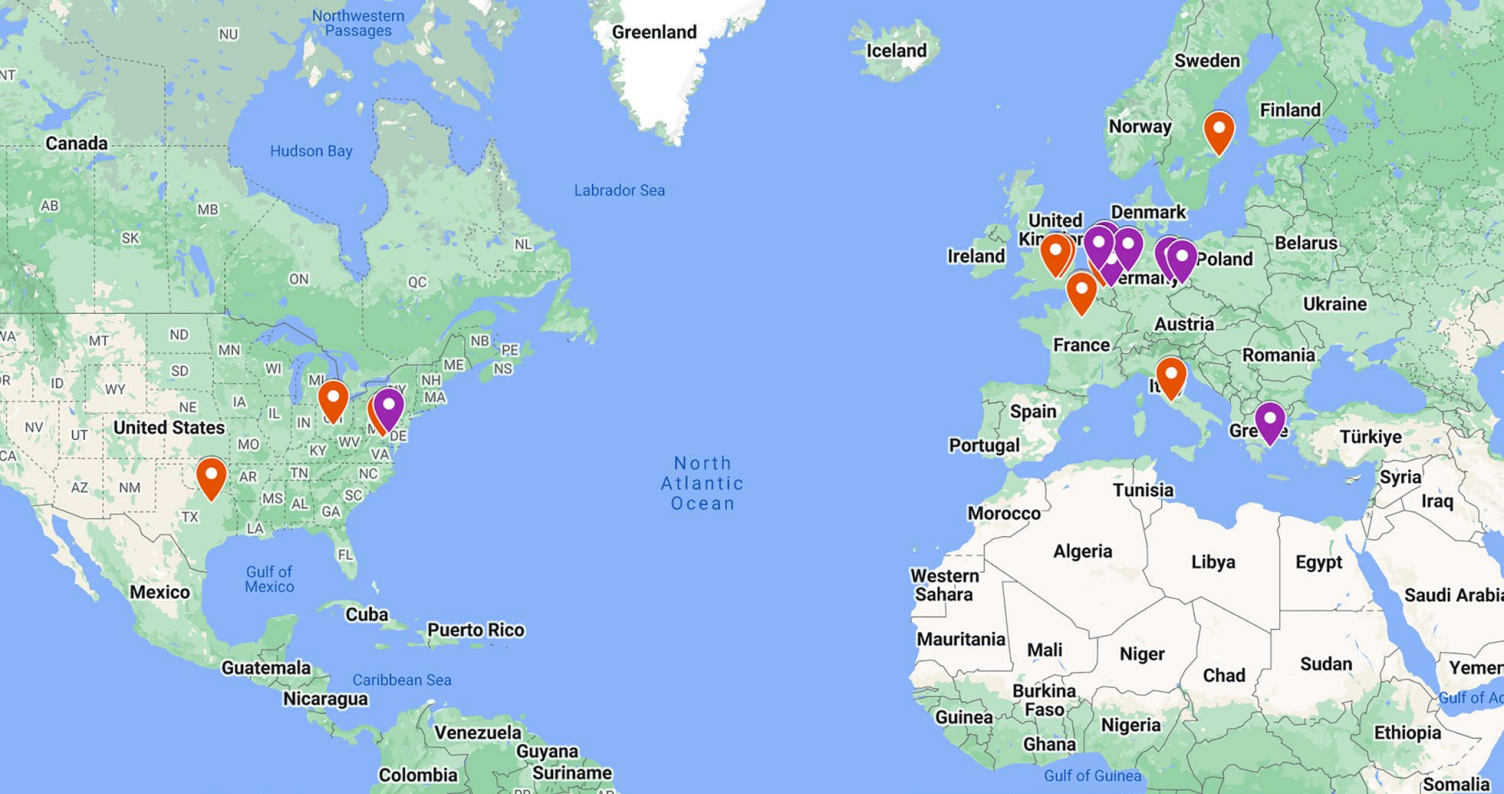
Sites currently performing iCMR procedures. Orange pins: iCMR diagnostic catheterization sites; Purple pins: iCMR electrophysiology sites. iCMR, interventional cardiovascular magnetic resonance. Map data ©2023 Google

iCMR diagnostic cardiac catheterization has been reported by different groups in pediatric and adult patients (Fig. 1). At several of these sites, iCMR catheterization is standard clinical practice for all patients requiring diagnostic hemodynamic studies. Clinical applications vary from simple right heart catheterization in patients with pulmonary hypertension, to more extensive hemodynamic studies in patients with complex congenital heart disease (Table 1). A key strength of iCMR catheterization is the ability to integrate phase contrast measurements of cardiac output (and differential pulmonary and systemic flows), which have been shown to be more reliable than thermodilution (which is imprecise and is limited by presence of tricuspid regurgitation) or Fick principle (which is usually calculated using estimated rather than measured VO_2_, and at rest rather than during other hemodynamic conditions) [1, 2]. Most procedures have been performed to date at 1.5 T field strength, using unmodified non-metallic balloon wedge end-hole catheters. Dedicated MRI-conditional non-metallic guidewires have been used in some centers [3, 4]. More recently, feasibility of using certain fully insulated nitinol guidewires has been demonstrated under low-SAR and low-field imaging [5, 6]. iCMR catheterization provides simultaneous pressure measurements (via the invasive catheter) and flow measurements (using phase contrast MRI). Sequences can be added to the clinical iCMR protocol to quantify cardiac chamber volumes and systolic function, screen for pulmonary emboli, measure lung water, and can be combined with physiological provocations (e.g. exercise or inhaled nitric oxide) to unmask latent symptoms or test for responsiveness to pharmacological therapy [7]. The ability to measure cardiac output during exercise is a particular strength of CMR, because Fick is unreliable without direct measurement of VO_2_ during exercise.

**Table 1:** Reported experience of iCMR cardiac catheterization procedures

Author	Year	Number of patients	Field strength (T)	Patient population	Comments
Razavi et al. [3]	2003	16	1.5	Pediatric and adult congenital	Non-metallic guidewire
Muthurangu et al. [19]	2004	24	1.5	Pediatric and adult congenital	
Kuehne et al. [99]	2005	15	1.5	Pediatric and adult	
Ratnayaka et al.[11]	2013	16	1.5	Adult	
Rogers et al. [20]	2017	102	1.5	Adult	
Ratnayaka et al. [8]	2017	39	1.5	Pediatric	
Campbell-Washburn et al.[5]	2018	7	1.5	Adult	Low-SAR imaging with nitinol guidewire
Knight et al.[12]	2019	50	1.5	Adult	
Campbell-Washburn et al. [6]	2019	7	0.55	Adult	Low-field imaging with nitinol guidewire
Veeram Reddy et al. [4]	2020	34	1.5	Pediatric and adult congenital	Non-metallic guidewire
Velasco Forte et al. [100]	2021	30	1.5	Pediatric and adult congenital	
Greer et al. [101]	2022	60	1.5	Pediatric and adult congenital	T1-overlay visualization technique

*iCMR* interventional cardiovascular magnetic resonance, *SAR* specific absorption rate

### Pediatric and adult congenital heart disease

iCMR catheterization is well suited for congenital heart disease patients. Indeed, this was the patient cohort described in the first CMR catheterization report [3]. Congenital heart disease is comprised of multiple complex structural heart anomalies with variable pre- and post-surgical anatomy. CMR imaging can guide invasive catheter navigation in complex anatomy. Invasive catheter pressure measurements coupled with CMR hemodynamics such as flow/shunt quantification to guide clinical management. Uniquely CMR allows measurement of flow in individual blood vessels, such as branch pulmonary arteries. For example, CMR catheterization data can determine operability of a patient with intracardiac shunt, which is the most common group of congenital heart defects. Pediatric non-congenital heart disease patients, such as those with pulmonary hypertension, with cardiomyopathy, or after heart transplant can also benefit. CMR catheterization is a radiation-free alternative for children, who are theoretically most susceptible to harmful effects of repeated ionizing radiation [8].

There is now greater than fifteen years’ experience performing CMR catheterization at a growing number of pediatric centers [4, 8–10] (Table 1). In a recent series, the first 50 consecutive pediatric CMR right heart catheterizations were all successful with real-time CMR guidance alone [8]. This included subjects (36%) with prior metallic implants that produce CMR imaging artifacts. There were no complications. With respect to radiation exposure, more than half of the subjects had undergone multiple (5.5 ± 5) prior X-Ray cardiac catheterizations. Similar to adult CMR catheterization teams [11, 12], with repetition procedure times for pediatric CMR catheterization can improve to approximately 15 min per hemodynamic condition [8]. Pediatric patients often require the added complexity of endotracheal intubation and general anesthesia. Nevertheless, with planning, practice and safety protocols almost all patients are safely managed [13].

### Radiofrequency ablation

**Fig. 2:**
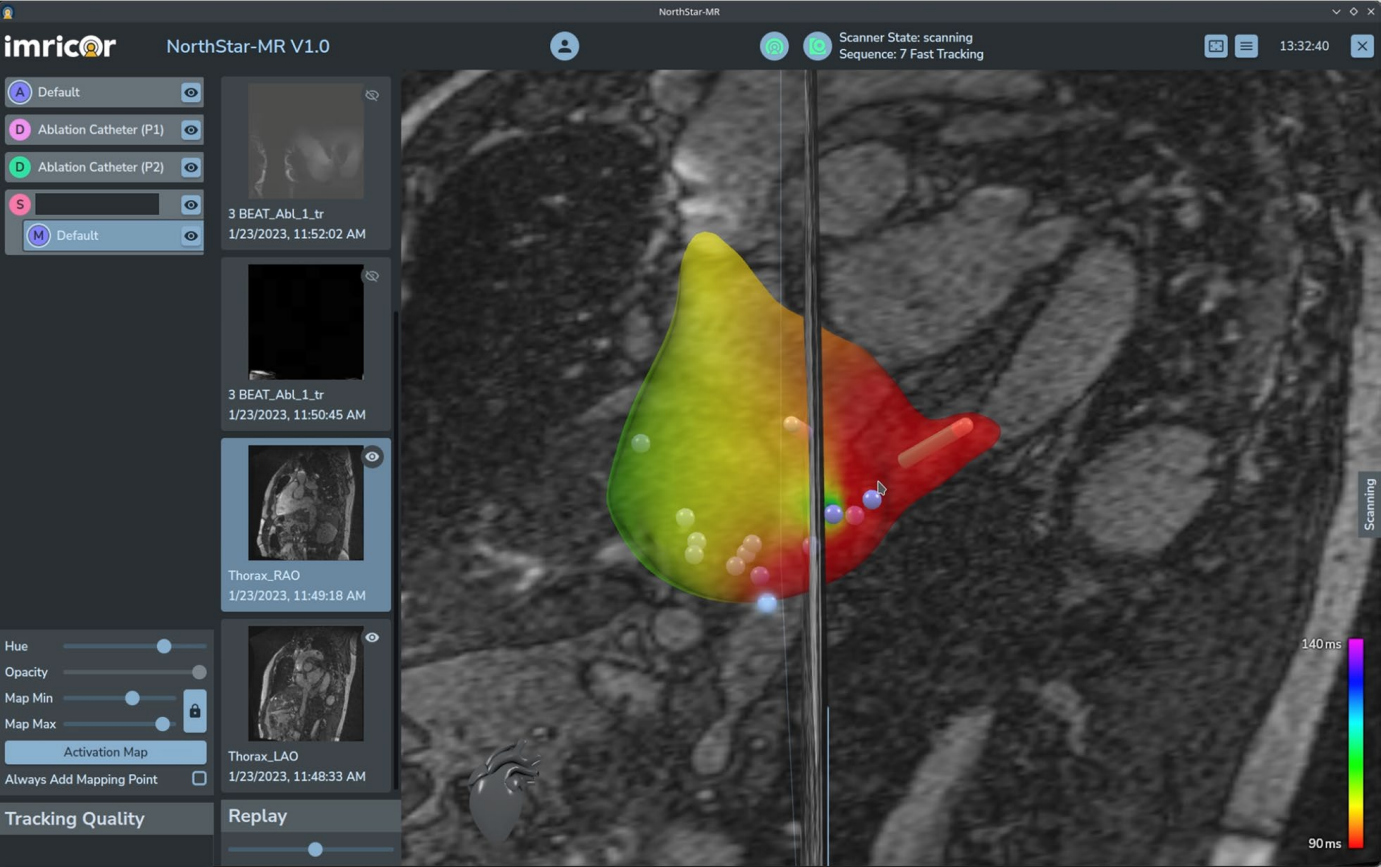
iCMR atrial flutter ablation. Activation map superimposed on anatomic MR images during CMR-guided atrial flutter ablation. Image courtesy of Ivo van der Bilt, MD and the MRI Ablation Center at Haga Teaching Hospital, Netherlands. CMR, cardiovascular magnetic resonance, iCMR, interventional cardiovascular magnetic resonance

Clinical ablation under iCMR guidance is now feasible (Table 2). Early human studies demonstrated iCMR guidance of non-ferromagnetic catheters to the right atrium and ventricle and making atrial, ventricle, and His bundle intracardiac electrogram recordings during real-time MRI scanning [14]. Commercial MR compatible EP catheters that incorporate heating mitigation components now permit more reliable active catheter visualization and position tracking [15, 16] and no longer restrict MR imaging to lower quality, low SAR pulse sequences [17]. Combined with commercial MR compatible electrogram recording and pacing equipment, patient studies have demonstrated multi-catheter atrial and ventricular pacing and electrogram recording, which is required for diagnostic EP studies [17]. Clinical ablation has been safely performed under real-time MRI guidance using a commercial MR compatible radiofrequency (RF) generator [15, 16, 18] (Fig. 2).

**Table 2:** Reported experience of iCMR electrophysiology procedures

Author	Year	Number of patients	Field strength (T)	Patient population	Comments
Nazarian et al. [14]	2008	2	1.5	Adult	Passive catheter tracking
Piorkowski et al. [102]	2013	1	1.5	Adult	Passive catheter tracking
Sommer et al. [17]	2013	5	1.5	Adult	Passive catheter tracking
Nordbeck et al. [103]	2014	1	1.5	Adult	Passive catheter tracking
Hilbert et al. [18]	2016	6	1.5	Adult	Active catheter tracking
Chubb et al. [16]	2017	10	1.5	Adult	Active catheter tracking
Paetsch et al. [15]	2019	30	1.5	Adult	Active and passive catheter tracking

*iCMR* interventional cardiovascular magnetic resonance

Beyond confirming basic procedure safety, effective iCMR guided ablation has been demonstrated for the simple arrhythmia, typical atrial flutter. Curing this arrhythmia requires anatomically guided ablation of the narrow isthmus of myocardium between the inferior vena-cava and tricuspid valve. Earlier iCMR guided atrial flutter ablation studies reported relatively poor outcomes, in part attributed to limitations in the MRI compatible EP catheter reach, not accounting for higher ablation power dissipation by heating mitigation components, lack of prior metrics for confirming adequate catheter-tissue contact, and limited operator experience in the unfamiliar iCMR environment [16, 18]. However, the most recent single-center series of 30 patients achieved an iCMR guided ablation success of 93%, which is comparable to conventional ablation outcomes for typical atrial flutter [15]. Procedure time, ablation time, and number of procedures to gain competence were also reported comparable to conventional ablation. Standardization of imaging protocols to generate MR images that are equivalent to the X-ray projection planes with which the electrophysiologist is familiar is useful [19].

## What infrastructure and personnel are needed to perform iCMR procedures?

### Is field strength important for iCMR procedures?

iCMR is technically demanding and requires rapid frame-rate imaging of the heart, vessels, and interventional devices. Technical specifications of gradient performance, magnetic field homogeneity, and coil array technology, as well as pulse sequence and image reconstruction capabilities allow contemporary MRI systems to meet the fast-imaging demands of iCMR. Other MRI system specifications such as bore dimensions (diameter and length), and patient transfer table design are also critical components to facilitate iCMR procedures.

iCMR cardiac catheterization has been reported at 0.55 T [6] and 1.5 T [3, 4, 8, 11, 12, 20, 21]. Most iCMR procedures are performed using 1.5 T due to image quality, ease of use, availability, and favorable properties for real-time imaging. While 3 T offers high SNR for intraprocedural imaging (e.g., lesion characterization), in practice 3 T imaging suffers from insurmountable problems of susceptibility artifacts and heating of interventional devices that contain metal, as well as hardware complexity and cost. Systems with lower magnetic field strengths (≤ 1.5 T) are attractive because they may allow use of off-the-shelf metallic devices, paired with high-performance scanner hardware suitable for iCMR.

### Why low field may be better for iCMR

The mechanical performance of most interventional devices is largely dependent on metallic components that are intrinsically conductive and therefore prone to significant heating and, in many cases, image distortion. Lower magnetic field strengths are inherently safer for metallic devices because RF power is proportional to the square of the main magnetic field. In addition, the RF wavelength is inversely proportional to magnetic field strength, meaning that lower field strengths require a longer conductive device to develop standing waves that result in RF-induced heating [22] (see section on iCMR device safety below).

**Fig. 3:**
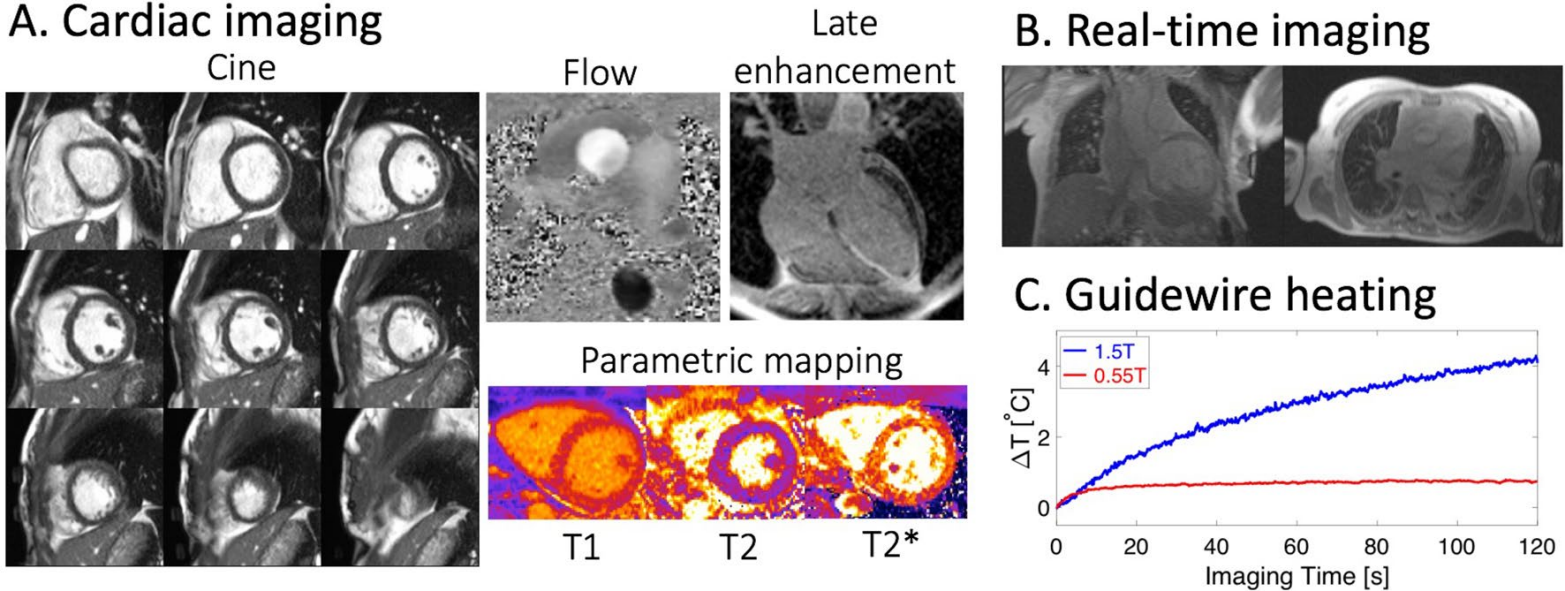
CMR at low field. **A** Example cardiac MR images acquired using a high-performance 0.55 T imaging system illustrating the retained image quality. **B** Real-time bSSFP imaging used for patient right heart catheterization at 0.55 T. **C** Guidewire (180 cm × 0.035″ Glidewire, Terumo, Tokyo, Japan) heating in an ASTM gel phantom measured at 1.5 T and prototype 0.55 T (both MAGNETOM Aera, Siemens Healthcare, Erlangen, Germany). *ASTM* American Society for Testing and Materials, *bSSFP* balanced steady-state free precession, *CMR* cardiovascular magnetic resonance

Early low field MRI systems, including open and double-donut systems at 0.2 T and 0.5 T, had inadequate hardware performance and gradient strength by modern standards, and thus were unsuitable for iCMR procedures [23]. Indeed ‘low field’ was historically interpreted to mean ‘low SNR’, but with modern hardware and software, loss of SNR can be compensated for by more effective use of available signal to obtain images that are indistinguishable from higher field scanners [6]. Figure 3 shows examples of attainable cardiac image quality using a prototype 0.55 T MRI scanner and we have previously described its potential value for imaging other organ systems [6]. Low field systems are cost-efficient to manufacture and therefore may be more affordable even if they are not being used all the time. A 0.55 T low-field 80 cm wide-bore MRI scanner is now commercially available. A prototype low-field scanner with high-performance gradients is under evaluation but is not yet commercially available.

### Do I need a co-localized X-ray catheterization laboratory?

Many teams performing iCMR procedures prefer suites with co-localized X-Ray fluoroscopy and MRI rooms [8, 9, 21]. Typically, the two rooms are separated by doors allowing for independent use and inter-modality patient transfer. This arrangement minimizes X-Ray or CMR room idle time and allows scheduling flexibility as iCMR case volume increases over time. It is attractive for ease of vascular access prior to iCMR catheterization and post-procedure recovery (e.g., hemostasis, extubation) in a familiar environment. The adjoining X-Ray room is available for rare but potentially emergency transcatheter bailout or patient cardioversion/defibrillation/pacing. In addition to iCMR catheterization, this configuration facilitates procedures that use the same encounter or (mostly in pediatrics) same anesthesia: X-Ray fused with CMR, non-invasive CMR to plan X-Ray procedure, post X-Ray procedure “exit” CMR, and X-Ray plus diagnostic MRI (cardiac and non-cardiac). Co-localization may be preferred when designing new construction and can be justified when planning to treat higher risk patients and perform interventional versus diagnostic iCMR catheterization. MRI hardware weight, vibration, and noise impact on neighboring rooms should be carefully considered during architectural siting.

More recently, iCMR teams have been successful performing iCMR catheterization in their existing, standalone MRI scanner [12]. This avoids the high cost of co-locating MRI and X-ray systems and allows teams to adopt iCMR procedures sooner. It also simplifies patient transfer with respect to invasive lines, sheaths, or catheters in potentially intubated patients. It does require contingency planning for emergency patient resuscitation outside the MRI room (space allocation) and an emergency evacuation strategy to X-Ray (room, staff availability). A standalone MRI room may be sufficient for low-risk invasive diagnostic iCMR catheterization. Even though heating, ventilation, and air conditioning (HVAC) systems in diagnostic MRI rooms typically do not meet requirements for invasive catheterization procedures, early adopters have successfully obtained institutional exemptions. Multidisciplinary collaboration (e.g., between cardiologists and radiologists) is desirable if contemplating performing iCMR procedures in an MRI room that is not set up for invasive procedures and is far from the cardiac cath lab.

## What personnel is needed for iCMR procedures?

The minimum personnel required for iCMR cardiac catheterization includes the MRI technologist to drive the scanner, the cath lab technologist to prepare interventional equipment, the cath lab nurse to monitor and administer conscious sedation, and the interventional cardiologist/electrophysiologist to manipulate catheters. In practice, many centers have additional staff depending on the procedure being performed or the patient population. An imaging cardiologist/radiologist with expertise in CMR is invaluable. In addition, pediatric interventional procedures are commonly performed under general anesthesia; electrophysiology studies require a technologist to drive the mapping and ablation system; and pulmonary hypertension hemodynamic studies may require a respiratory therapist to administer inhaled nitric oxide.

## What other equipment do I need to get started?

Table 3 summarizes key equipment required to start an iCMR program. Some components are necessary, and others are optional but highly desirable to ensure that the user experience is similar to an X-ray cardiac catheterization laboratory. Not all equipment is commercially available and so in practice, many centers classify iCMR procedures as research. All equipment entering the MRI room must be evaluated for MRI safety. A globally recognized schema classifies objects and medical devices as MRI unsafe, MRI conditional, or MRI safe (Table 4). A comprehensive and searchable list of medical implants is available online (https://www.mrisafety.com).

**Table 3:** Equipment “shopping list” to start an iCMR program

	Necessary	Desirable
MRI scanner	▪ 1.5 T high performance MRI system	▪ Low-field MRI system (high performance 0.55 T iCMR system currently in development)
MRI scanner interface	▪ Real-time CMR and interactive user interface (available from most MRI system vendors)	
Communication	▪ MRI system patient headset for the in-room operator to wear to communicate with MRI technologist in the control room	▪ Noise cancelling, multi-channel staff communications system for safe operation despite the extreme acoustic noise around the scanner (representative example: Optoacoustics, Yehuda, Israel) [8, 20]
Video displays	▪ In-room shielded video projector or monitor to display at a minimum real-time images and hemodynamics for in-room operators	▪ Multiple monitors/projectors or single monitor with multiple image inputs to display real-time CMR images, invasive pressure waveforms, ECG, pulse oximetry ideally at both head and foot of the patient bed
Hemodynamic monitoring	▪ Hemodynamic monitoring system (low-fidelity, existing anesthesia MRI patient monitor)	▪ Hemodynamic recording system (high-fidelity, X-Ray catheterization lab equivalent)
		▪ A system to connect electrocardiogram and invasive blood pressure signals during MRI into a high-fidelity hemodynamic recording system, for example PRiME (Physiological Recording in MRI Environment) [24]
Anesthesia equipment	▪ Not required for procedures in adults using local anesthesia/moderate sedation	▪ For pediatric iCMR procedures, MRI-conditional ventilator/anesthesia cart for general anesthesia is often required [13]
Catheters	▪ Non-metallic balloon wedge end-hole catheters for diagnostic iCMR catheterization (Fig. 4)	▪ MRI-conditional electrophysiology recording and ablation system
Guidewires		▪ Fully insulated nitinol-core guidewires may be used under low-SAR imaging or in low-field MRI system ▪ Non-metallic MRI-conditional guidewire (0.035" EmeryGlide, B. Braun, Bethlehem, PA) ▪ “Active” antenna-guidewires
General supplies	▪ General procedural supplies are similar to in an X-Ray environment: for example, patient sterile draping [98], syringes, fluid-filled tubing and pressure transducers, etc.…	
Emergency bail out/defibrillation	▪ Defibrillator in a room outside the MRI suite to which the patient can be evacuated in an emergency	
	▪ Standard operating procedures and regular evacuation drills can optimize safety and efficiency [12, 13]	

*iCMR* interventional cardiovascular magnetic resonance, *SAR* specific absorption rate

**Table 4:** MRI safety classification

Classification	Description
MRI unsafe	Object may not enter the MRI room under any circumstances. Patients with MRI unsafe devices should not be scanned
MRI conditional	Object may enter the MRI room only under the specific conditions provided in the labeling. Patients with MRI conditional devices should not be scanned unless the device can be positively identified as MRI Conditional AND the conditions for safe use are met Conditions for use may include restrictions regarding: • Static field strength • Maximum spatial field gradient • dB/dt limitations (usually only applicable to active implants) • SAR limits • Other conditions, for example restrictions on the types of coils that may be used MRI Conditional objects NOT intended to enter the bore of the MRI scanner must typically be tethered or affixed to an unmovable part of the room
MRI safe	Object poses no safety hazards and may be placed anywhere in the MRI room. Patients with MRI Safe devices can be scanner without restrictions

Adapted from ‘Understanding MRI Safety Labeling’ (https://www.fda.gov/media/101221/download)

*SAR* Systemic absorption rate

## How do I monitor the patient during iCMR procedures?

Managing patient hemodynamics during cardiovascular interventions can be divided into either monitoring or recording. Monitoring focuses on patient safety during catheterization, including non-invasive pressure, end-tidal CO_2_, and peripheral hemoglobin oxygen saturation. Such monitoring systems are commercially available, but do not have the functionality of recording systems for obtaining high-fidelity cardiovascular measurements of invasive pressure traces and electrocardiography (ECG) for quantitative measurements.

Recording ECG during MRI is non-trivial due to signal perturbation from blood ions flowing through the main magnetic field (the magnetohydrodynamic effect, MHD) and from the imaging gradients. Commercial MRI systems use 3–4 ECG leads for cardiac triggering, and though the in-built filtering is robust, these tracings are not suitable for advanced diagnostics (e.g., interpretation of ST segment changes). Custom solutions have been developed using adaptive filtering based on the imaging gradient hardware for high-fidelity hemodynamic recording [14, 24, 25], and these corrected signals can additionally be used for appropriate triggering of MRI acquisitions. One of these is available as open-source plans that can be manufactured on-demand by sites (https://nhlbi-mr.github.io/PRiME/). To enable use of this custom solution in patients, many centers classify iCMR procedures as research.

Preferably iCMR would employ identical monitoring and recording systems as X-ray. However, conventional X-ray surface electrodes are typically connected to long, conductive transmission lines that are unsuitable for MRI due to induced heating and electrical noise. As dual-use ECG transmission lines are not commercially available, most interventional labs use two sets of leads, which comes at an inconvenience cost during inter-modality patient transfer [26].

## How do I obtain reliable electrocardiograms?

Surface ECG monitoring is used to screen for arrhythmias and cardiac ischemia during conventional cardiac procedures and is similarly desirable for iCMR guided procedures. For cardiac electrophysiology (EP) procedures, the ECG also helps localize arrhythmia origin to guide more detailed invasive diagnosis using intra-cardiac electrogram measurements.

The MRI environment presents several challenges to acquiring high-fidelity ECG and electrograms. MRI RF transmission can induce currents in elongated conductors, such as ECG lead cables and catheters [22, 27]. The resulting risk of significant heating has been mitigated by high impedance wires for ECG and electrogram recording [28, 29] and wireless transmission systems that permit use of short ECG leads [30]. MRI also uses time-varying spatial gradients of the scanner magnetic field for image generation. This induces currents in conductor loops, such as between ECG leads, which can overwhelm cardiac electrical signals. Low pass filtering is helpful for mitigating this noise [14]. Because noise frequencies have some overlap with ECG signals, however, additional filtering strategies have been used including adaptive filtering and briefly blocking electrogram recording when currents are induced [24, 31]. Even in the absence of imaging, blood flowing perpendicular to the strong MRI static magnetic field generates a voltage through the MHD. The MHD significantly obscures the surface ECG ST segment, which is important for assessment of cardiac ischemia, and can also affect QRS complex interpretation. Current MRI ECG monitoring systems have focused on detecting gross QRS complex timing rather than preserving ECG features for diagnostic interpretation. Strategies for filtering electromagnetic interference and MHD that preserve diagnostic information for 12-lead ECG inside the MRI scanner have been described but challenges to clinical implementation remain [24]. Since MHD increases with magnetic field strength, lower-field MRI may improve ECG interpretation within the scanner [6]. Because of the small spacing between electrodes and electrode wires, MHD has little impact on the bipolar electrogram recordings but needs to be considered for unipolar electrograms.

## Unique safety precautions for iCMR

Procedures in an interventional CMR suite require adjustments to traditional catheterization and EP lab routines. Multidisciplinary staff require education and specialized safety training prior to participation in MRI procedures. Patients undergo MRI safety screening at their pre-procedure appointment and again on arrival to the lab. Typically, vascular access is obtained outside the MRI room (e.g., in an adjacent X-ray suite or procedure room). It is good practice to perform a sharps and metal “time out” with at least two staff members prior to transfer into the MRI room to ensure the removal of ferromagnetic objects from the sterile field and to avoid transporting MRI-unsafe devices from the X-ray cath lab into the MRI room. Draping maintains the sterile field during transfer into the CMR scanner room and throughout the iCMR procedure. Dedicated noise-cancellation headsets with microphones enable communication between team members and the patient and suppress the noise of the MRI scanner.

## What happens in an emergency?

Life-threatening hemodynamic instability that necessitates defibrillation, cardioversion, or emergency pacing; or pericardiocentesis in the event of cardiac tamponade, cannot be treated within the MRI suite at present. CMR guided procedures require protocols and regular training for rapid patient transfer from the MRI scanner to a designated location containing a defibrillator and other MRI Unsafe resuscitation equipment (e.g., oxygen cylinder, bronchoscope, or ventilator). Simulated emergency transfer drills are important for staff to familiarize themselves with role-specific tasks and for the team to identify potential pitfalls. Labs performing iCMR procedures train on a regular basis targeting an evacuation time of 1 min [8, 21].

To the best of our knowledge, there is no commercially available MRI-conditional temporary transvenous pacemaker. In the event of hemodynamically compromising heart block, most centers transfer the patient to outside the MRI room for temporary transcutaneous pacing or to the X-ray cath lab for temporary transvenous pacing. Currently, the only way to perform transvenous pacing in the scanner is using the ablation catheter provided with an MRI-conditional mapping and ablation system [15]. For patients with heart block who need to undergo MRI, feasibility of temporary pacing using a conventional MRI-conditional pacemaker and lead with the box taped to the outside of the patient’s chest has been reported [32].

## How do I perform defibrillation?

When patients undergoing MRI require defibrillation, current practice is transfer outside the scanner area before performing defibrillation with conventional defibrillator equipment. This practice is suboptimal for interventional procedures since prolonging time to defibrillation reduces success [33], and multiple defibrillations may be needed during a procedure.

MRI-conditional defibrillators are in development but not yet commercially available [34]. These modify commercial defibrillators with output wire filters designed to (1) suppress defibrillator electrical noise that would corrupt MR images, (2) suppress radio-frequency currents generated during MRI scanning that could lead to heating of patient defibrillator electrode patches, modified for safety, and (3) tolerate high voltages generated by the defibrillator. Safe scanning with acceptable levels of electrode heating and image noise has been demonstrated in human volunteers with connected defibrillator patches and successful defibrillation has been performed in animals with ventricular fibrillation [34, 35].

## Why do iCMR devices need to be different?

Under standard real-time MRI, interventional devices such as catheters, guidewires, stents, and balloons, which contain metal cores or braiding, may create artifacts on the MRI images that obscure the target anatomic structures or others may be nearly invisible. Furthermore, metal-containing devices may heat from the RF energy delivered during MRI imaging. Plastic/polymer only catheters are intrinsically safe within the MRI environment, but their mechanical properties are limited. Thus, catheter devices must be re-engineered to assure MRI safety and optimal visualization, while maintaining the necessary mechanical properties for the intended application. As described previously, switching to low-field MRI allows for some existing metallic catheters and guidewires to be used safely and without modification.

## How can iCMR devices be modified for safety?

The strong magnetic field employed in MRI systems induces displacement forces and/or torque on ferromagnetic materials. Thus, the first safety criterion for iCMR catheter devices is confirmation they do not comprise ferromagnetic materials. Long metallic structures (e.g., guidewires or braided catheters) are susceptible to radiofrequency (RF) energy-induced heating which may potentially cause burn-related injury. Alternating currents induced on metallic objects during the RF excitation, cause an accumulation of electrical charges generating standing waves on the device. The electric field and therefore heating typically concentrates at the tip of the device [36]. The amount of heating depends on multiple parameters including magnetic field strength, flip angle, device length, presence or absence of insulation on the device, insertion length into the body and the position of the device in the body and within the MRI scanner bore. Maximum heating occurs when the electrical length of the device is equal to the half wavelength of the Larmor frequency (~ 37 cm for 0.55 T, ~ 12.5 cm for 1.5 T, ~ 7.5 cm for 3 T systems). However, the effective wavelength of devices changes significantly with the insulation thickness, loading condition, insertion length, etc. So, both device configuration and conditions of use need to be considered when evaluating a specific device. Several methods have been proposed to limit or minimize RF-induced heating of iCMR devices, including detuning the device during RF transmission [37], use of RF chokes [38] or transformers [39] on transmission lines, using high resistance wires as transmission lines [29], replacing transmission lines with fiberoptic connections [40] and segmenting conductive components shorter than quarter wavelength [41]. Also, special MRI sequences have been developed to reduce the amount of transmitted RF power to eliminate RF-induced heating during MRI scans[5] (see section on pulse sequence adaptation below).

## Guidewires for iCMR

Interventional cardiovascular procedures require guidewires as part of the basic tool kit, permitting safe passage of interventional devices such as catheters and angioplasty equipment to their intended target. Manufacturers construct wires with the aim of creating an optimal balance of pushability, torquability, trackability and visibility. Metal is used in most guidewires designed for use in X-ray to provide optimal mechanical performance, but this makes them particularly susceptible to RF-induced heating and magnetic susceptibility artefacts if used in MRI. Commercially available, fully insulated nitinol wires (e.g., Glidewire, Terumo, Tokyo, Japan) have been used with success at 1.5 T under very specific imaging parameters [5]. Nitinol’s paramagnetic properties result in a modest signal void which can be exploited using techniques such as positive contrast imaging with real time color overlay to increase visibility [42]. Alternatively, new 0.55 T low field scanners should enable right heart catheterization using unmodified off-the-shelf insulated nitinol guidewires [6].

Purpose built guidewires have been designed and manufactured to overcome some of the problems faced by off the shelf devices at 1.5 T. Utility of a commercially available entirely non-metallic MRI-conditional guidewire with passive markers at the tip has been reported in pediatric and adult patients with congenital heart disease [4]. Other wires in development but not yet commercially available include the United States National Heart Lung and Blood Institute (NHLBI) passive guidewire which incorporates a segmented design, reducing RF induced heating by a factor of 10 compared to a 260 cm solid core nitinol guidewire [43]. Each nitinol segment is kept shorter than ¼ wavelength of the Larmor frequency at 1.5 T to prevent standing wave formation. Conspicuity is provided by the inclusion of iron oxide powder markers. An active guidewire, in commercial development in collaboration with NHLBI (*TRACR*, Transmural Systems, Andover, Massachusetts), uses a modified loopless antennae to ensure conspicuity of the guidewire along its length [44] (see section on device visualization below).

## Catheters for iCMR

**Fig. 4:**
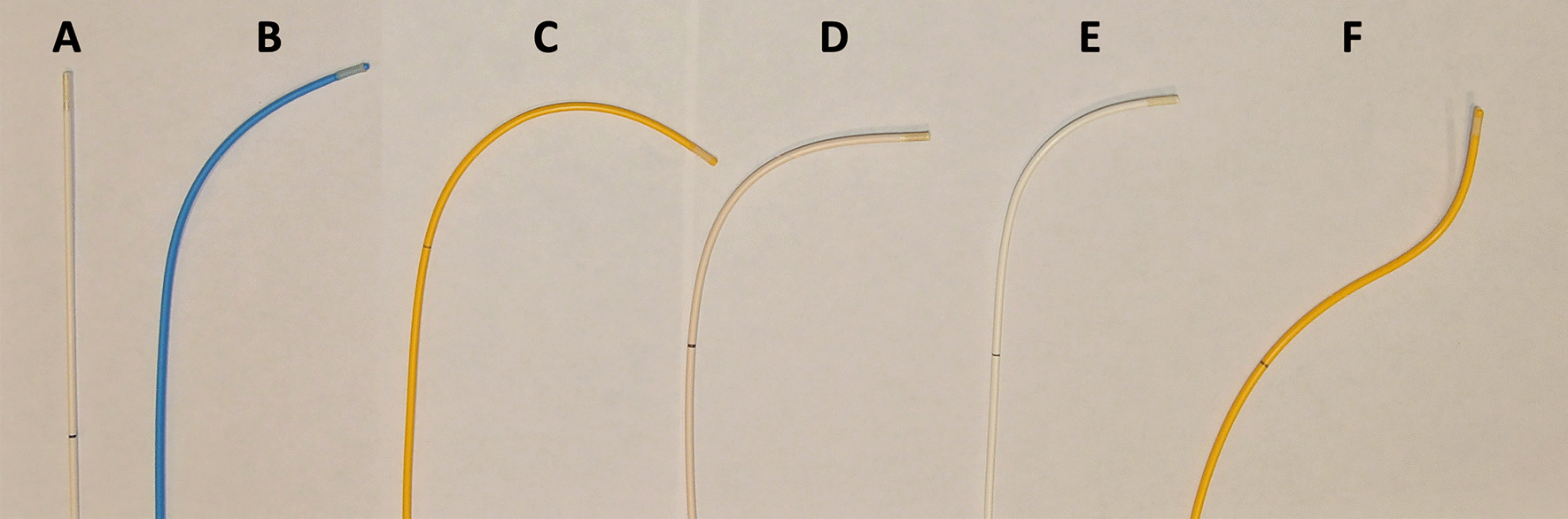
Commercially available non-metallic catheters for iCMR catheterization procedures. **A** Flow Directed Balloon Catheter, Cook, Model #: FDB5.3-35-80 (Cook, Bloomington, Indiana, United States); **B** Pulmonary Wedge Pressure Catheter, Model #150075 (Medtronic, Minneapolis, Minnesota, United States); **C** True Size Monitoring Catheter, Model #111F7 (Edwards Lifesciences, Irvine, California, United States); **D** Edwards, True Size Hi-Shore Monitoring "T" Tip Catheter, Model #T111F7 (Edwards); **E** Balloon Wedge Pressure Catheter, Model #AI-07127 (Teleflex, Wayne, Pennsylvania, United States); **F** True Size Monitoring “S” Tip Catheter, Model #S111F7 (Edwards). *iCMR* interventional cardiovascular magnetic resonance

Balloon wedge end-hole catheters without metallic braiding, the workhorse catheter for diagnostic right heart catheterization under x-ray fluoroscopy, have been used safely and effectively in over a thousand patients in the MR environment with the balloon-tip filled with gadolinium or air (Fig. 4). Gadolinium based T1 shortening agents or air (or CO_2_) are used to inflate the catheter balloon for purposes of increased conspicuity under real time imaging [45, 46]. Gadolinium is preferred by some operators due to the frequency of other signal void artefacts and reduced certainty with which an air-filled balloon can be distinguished [11]. Operators should beware that substituting fluid for gas reduces tactile feedback during balloon inflation; inflation should only be performed under real-time imaging in situ to avoid vascular injury or rupture.

Off the shelf catheters, both braided and non-braided, have been used in the pre-clinical environment for left heart and great vessel catheterization. Stainless steel braid can be problematic due to magnetic deflection, heating, and susceptibility artefacts at all field strengths. Those constructed of ferromagnetic 304 stainless steel cause significant artifact on MR images. In contrast, 316L has negligible magnetic attraction and negligible signal void artefact during real time MRI.

As with guidewires, attempts have been made to construct purpose-built devices to circumvent visualization and safety issues encountered with off the shelf devices at commonly used field strengths. Pre-clinical procedures have been performed with these promising prototypes, but clinical translation remains elusive [47, 48].

### Mapping and ablation catheters for iCMR

Electroanatomic mapping keeps track of the 3D anatomic location of electrogram measurements and ablation delivery and is widely used in current EP practice. Equivalent mapping is desirable for iCMR guided EP procedures. Commercial mapping systems detect catheter position relative to applied electric and/or magnetic fields [49]. Though commercial magnetic-field based tracking systems are incompatible with the MRI environment, use of electric-field based mapping inside the MRI scanner has been demonstrated [50]. Mapping catheter location can also be determined from small imaging coils incorporated into the device [51, 52]. MRI based catheter tracking has the advantage of inherent registration to myocardial scar, fibrosis, and ablation lesion imaging which are the main motivation for performing iCMR EP procedures. First generation MRI-conditional mapping systems are being used by sites currently evaluating clinical iCMR guided EP procedures [15–18].

In addition to the MRI compatibility considerations described above, EP interventional devices have specific heating safety considerations. EP catheters typically contain multiple long wires connected to distal electrodes for measuring intra-cardiac electrical signals, delivering ablation energy, and transmitting catheter position information which could lead to device heating. High resistance wire is effective for mitigating the heating risk of electrodes used to measure intra-cardiac electrograms and impedance-based mapping system signals [28, 29]. Transmission lines that utilize inductive coupling between shorter segments are effective for reducing the heating risk of catheter position tracking coils [39]. In cases where wires must efficiently transmit power, such as for radiofrequency (RF) ablation, high frequency RF filters can attenuate MR generated RF energy, while allowing passage of lower frequency RF ablation energy [28, 38, 53].

Commercial MRI conditional quadripolar EP catheters are now available and have been safely applied in human diagnostic EP and ablation studies [15–18]. Multi-electrode catheters for more efficient high-resolution mapping, standard tools for complex arrhythmia mapping, are in development for iCMR [54]. Progress is also being made toward improving the mechanical characteristics of MRI conditional EP devices. The non-metallic components often used to construct MRI conditional catheters reduce torque transmission and are less able to return to the same shape after catheter deflection. In addition, heating mitigation components occupy space within the catheter lumen resulting in larger diameter, stiffer devices. These limitations may be addressed by placing miniature “baluns” along the outside of metallic braided devices [55]. This method appears to adequately suppress current induction and heating along metallic braids and wired internal components during MRI scanning, avoiding need for heating mitigation components inside the catheter.

## How are imaging requirements for iCMR different?

### Real-time imaging

**Fig. 5:**
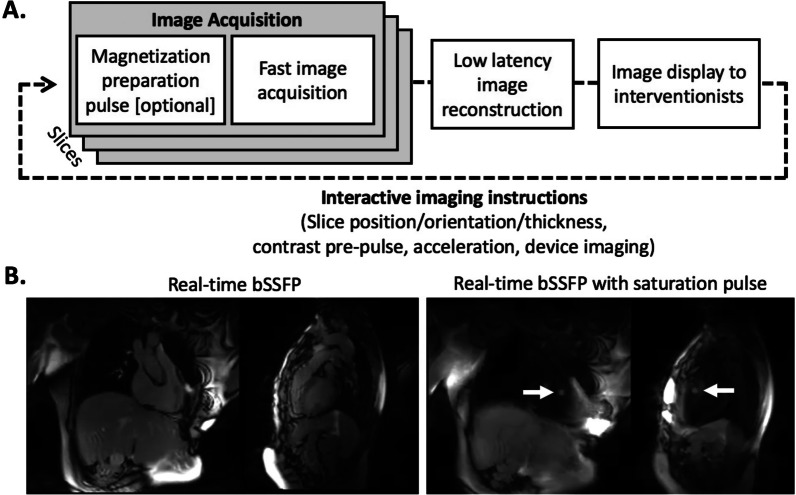
Real-time CMR. **A** Schematic diagram of real-time MRI fluoroscopy sequences for multi-planar interactive imaging. **B** Real-time bSSFP images with and without interactive saturation (“magnetization preparation”) pulses for gadolinium-filled balloon visualization from 1.5 T (MAGNETOM Aera, Siemens Healthcare, Erlangen Germany). Typical commercial real-time MRI parameters are 5–10 frames per second using bSSFP at 1.5 T (TE/TR = 1.3/2.6 ms, matrix = 192 × 144, acceleration factor = 2–4). bSSFP: balanced steady-state free precession

Real-time MRI can be used to navigate devices and guide invasive catheterization procedures. Real-time MRI demands high frame rates > 5 fps, interactive imaging modules, and visualization of both tissues and interventional devices. Operators require interactive control of slice position/orientation, slice thickness, acceleration rate and image contrast during MR fluoroscopy (Fig. 5).

**Fig. 6:**
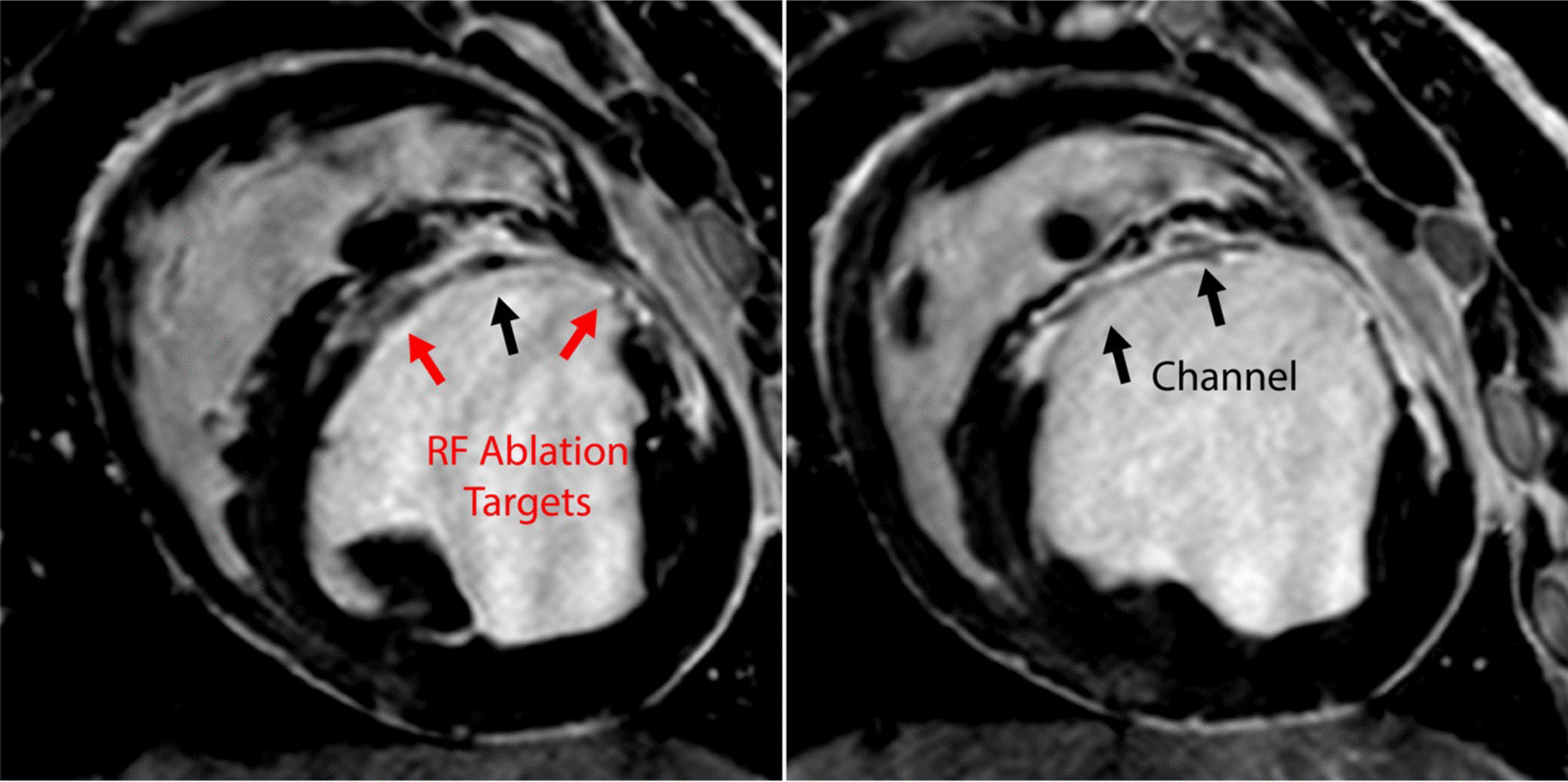
Scar imaging. High-resolution 3D late gadolinium enhancement (LGE) imaging demonstrates heterogenous myocardium in swine 2 months post-myocardial infarction. Regions of tissue heterogeneity (red arrows) and viable tissue channels traversing scar (black arrows) both represent targets in the treatment of ventricular tachycardia. This animal was inducible for ventricular tachycardia. *RF* radiofrequency. All animal experiments performed according to institutional guidelines

Most commonly, efficient multi-plane two-dimensional balanced steady-state free precession (bSSFP) imaging is used for fast image acquisitions with good blood-tissue contrast. bSSFP is combined with magnetization preparation pulses in order interactively to adjust imaging contrast to highlight specific features, such as gadolinium-filled balloons or pathological targets that enhance with gadolinium (Fig. 6) [46, 56, 57]. Faster frame rates can be achieved by adding advanced mathematical reconstruction techniques (examples include sensitivity encoding (SENSE) or generalized autocalibrating partially parallel acquisition (GRAPPA) and are referred to as ‘acceleration factors’ of 2 or more). More recently, machine learning algorithms have been used to remove artifacts in real-time diagnostic imaging [58], and we anticipate that machine learning will also find applications to augment and improve real-time iCMR. Real-time interactive sequences are available with most MRI vendors, and higher frame-rate imaging, specific image contrast modules, and device visualization methods continue to evolve.

### How can we improve device visualization?

Visualizing cardiovascular anatomy is straightforward using real-time MRI. By contrast, visualizing catheter devices using real-time MRI remains a challenge. “Passive” devices are visualized thanks to their intrinsic material properties. This approach is attractive because it is simple and safe but suffers from poor conspicuity. For example, balloon-tip catheters filled with gas (e.g., air or CO_2_) create signal voids, appearing as dark spots in the image [59]. In contrast, the same balloons filled with T1 shortening agents (e.g., gadolinium) [60] appear as bright spots, and can be further enhanced using full- or partial “saturating” magnetic pre-pulses to distinguish the device from surrounding tissue [46], which we too have adopted as our workhorse MR fluoroscopy pulse sequence. Some metallic devices can be visualized using the susceptibility artifact created by the device itself. Artifact size can be tuned on the device-design side by adjusting the iron content, and on the scanner side by varying the echo time, TE, during spoiled gradient echo acquisitions, at the expense of blood-myocardium contrast compared with bSSFP. Susceptibility-based device visualization can suffer when the susceptibility artifact is indistinguishable from other nonspecific dark spots (contrast signal voids), or when large susceptibility artifacts interfere with the assessment of surrounding tissues. To overcome this limitation, some devices rely on metallic markers capable of providing a unique imaging signature, such as known spacing between markers [12]. All imaging strategies for passive devices suffer from visualization loss as the devices move out-of-plane.

“Active” devices employ MRI receiver coils and transmission lines directly connected to MRI scanner and act as RF antennae for imaging, tracking or both. Two approaches are commonly applied: device profiling and device micro-coil tracking. Device profiling incorporates the device image in real-time images; this is the visualization approach used in X-ray fluoroscopy. Active devices can provide tip and whole shaft visibility on the image because of their high near-field SNR profile [61]. The active device signal received from a separate channel can be artificially highlighted and superimposed on the image. Since the device signal is directly carried to the scanner, active devices provide the highest device SNR, however tethered connection limits operational freedom. By virtue of incorporating long conductive wires, such devices are often prone to RF-induced heating [38]. The second approach, device tracking, uses projection acquisitions in three orthogonal planes to localize point-source signals from microcoils in order to provide device position and orientation information and used to overlay a synthetic image of the device position onto previously acquired images; this is the visualization approach popularized in electroanatomic mapping systems used in cardiac EP procedures.

Finally, semi-active devices incorporate resonator circuit elements and interact with the scanner via inductive coupling. These elements wirelessly couple to outside surface coils and serve as local RF signal amplifiers. They excite the surrounding tissue with a higher flip angle and appear as bright spots on the image. They do not require long transmission lines and may eliminate RF induced heating risk depending on the electrical length of the device. Such markers are typically more easily distinguishable compared with passive markers [62]. The conspicuity of semi-active devices typically diminishes with the increasing flip angle; however, this problem can be solved by exploiting the reverse polarization concept [63].

### How can we measure cardiac output using iCMR?

Interventional CMR can have significantly improved diagnostic value when the assessment of patient hemodynamics is paired with quantitative ventricular function and cardiac output measured by cine and phase-contrast imaging. Furthermore, as many patients with cardiopulmonary disease are asymptomatic at rest, a comprehensive diagnosis can be sought by quantitative recording across physiologic conditions such as exercise [64], fluid volume [65] or pulmonary vasodilation with inhaled nitric oxide [8].

Phase-contrast CMR is a robust and non-invasive measurement of cardiac output and has been shown to combine with pressure-catheter measurements to determine pulmonary vascular resistance, a strong prognosticator for pulmonary hypertension [1]. Phase-contrast images are typically acquired over a few minutes; the data are retrospectively sorted using the ECG-triggering in to a high spatial- and temporal-resolution datasets. However, ECG-triggering is unreliable during exercise, and so one method is to use the filtered pressure transducer waveforms for triggering. In the future the ideal approach may be to use a real-time pulse sequence to obtain a continuous readout of cardiac output rather than relying on patients’ ability to maintain exercise at steady state for the duration of conventional phase-contrast acquisition.

### When should pulse sequences be adapted to minimize SAR?

A different approach to mitigate RF-induced device heating is to modify the pulse sequences used during iCMR procedures. Device heating is related quadratically to B1 + and is inversely proportional to TR (defined as time between RF pulses). That is, high-power RF pulses and short durations between RF pulses result in increased device heating. The B1 + of a specific RF pulse depends on the pulse width and pulse amplitude. In general, real-time bSSFP uses a short sinc RF pulse for imaging efficiency, and device heating varies with the square of the flip angle. Magnetization preparation pulses used during real-time imaging, such as saturation pulses and inversion pulses, also contribute to device heating by deposition of RF power.

Previous work has manipulated pulse sequence parameters of real-time MR fluoroscopy to limit RF-induced heating by using spiral gradient echo imaging, which permitted safe use of a single commercial metallic guidewire (150 cm × 0.035″ Glidewire, Terumo, Tokyo, Japan) for right heart catheterization in patients [5].

Other pulse sequences used for assessing function, flow, and performing tissue characterization during an iCMR procedure can also contribute to device heating. Turbo spin echo (TSE) is an example of a high-SAR sequence used for black-blood cardiac imaging that may be inappropriate for application with a device in situ. We caution readers to assess heating from all pulse sequences prior to imaging with a metallic device in situ.

### How can we exploit CMR tissue characterization to guide procedures?

One requirement for more complex iCMR procedures is discrimination between the multiplicity of tissues during intervention. For example, for electrophysiological intervention in the treatment of arrythmias, pre-existing scar or fibrotic substrate and isthmuses present targets for therapy and identification of the extent and location of effected necrotic tissues is critical for assessment of procedural completeness [66]. Similarly, for successful MR-guided biopsy, guidance to regions of suspected disease (e.g., tumor, myocarditis, infiltrative disease, etc.) is necessary to avoid potential miss.

CMR can provide desired discriminatory information well beyond morphology and spatial localization, making it an excellent option for real-time targeting specific tissue types. Contrast can be achieved using both native tissue properties (primarily based on the relaxation rates T1 and T2 of tissue) as well as by the addition of differentially localized contrast-enhancing materials such as Gadolinium-based agents.

#### Scar imaging

Late gadolinium hyperenhancement (LGE) imaging is an excellent method to establish tissue viability as it can depict the extent and distribution of existing scar and fibrosis within preexisting arrhythmogenic substrate. Diagnostic LGE typically utilizes 2-dimensional (2D) imaging to derive structural prognostic indicators such as location and extent of scar, and degree of transmurality among many others. Although these quantities have diagnostic value, they may be insufficient to guide intervention. Hence, iCMR can benefit from pre-acquired higher image resolution roadmaps (~ 1 mm^3^ pixels) co-registered to real-time images during the procedure. However, static overlays have limitations. Ideally co-registered images would be dynamic and synchronize with cardiac and respiratory motion. Three-dimensional (3D) imaging has demonstrated better visualization of scar though more advanced methods of motion compensation are required and scan times are longer (Fig. 5). New approaches using pre-acquired and processed MRI roadmaps for guidance during intervention may enhance accuracy for guidance in the delivery of therapy or interrogation of particular tissue types.

#### Radiofrequency ablation lesion visualization

One reason why iCMR is attractive for EP procedures is the tantalizing potential to distinguish pre-existing scar from effective RF ablation lesions, and edema. The “holy grail” is to be able to interactively assess ablation completeness in both ventricles and atria. Originally, gadolinium-based agents were used to highlight ablation lesions using the same techniques as scar imaging. However, this approach results in uncertainty as many tissues enhance in a confounding manner (e.g., pre-existing scar, new RF ablations lesions and edema). Furthermore, enhancement of edema resulting from ablation results in overestimation of necrotic lesion volumes [67, 68], and due to dose limitations, gadolinium-based agents can be used once or twice during an intervention, further reducing utility for procedural guidance and assessment. Ablation lesion imaging is particularly challenging using real-time imaging in the thin-walled atria, particularly at low-field.

Temperature mapping (thermography) has also been used to monitor delivery of RF ablations [69–71]. The heat dose delivered through RF is estimated and the region of necrotic tissue is predicted. However, thermography is quite sensitive to non-homogeneities of the magnetic field, and RF ablation catheters and other devices needed during intervention and difficult post processing requirements can make it hard to estimate lesion extent accurately.

**Fig. 7:**
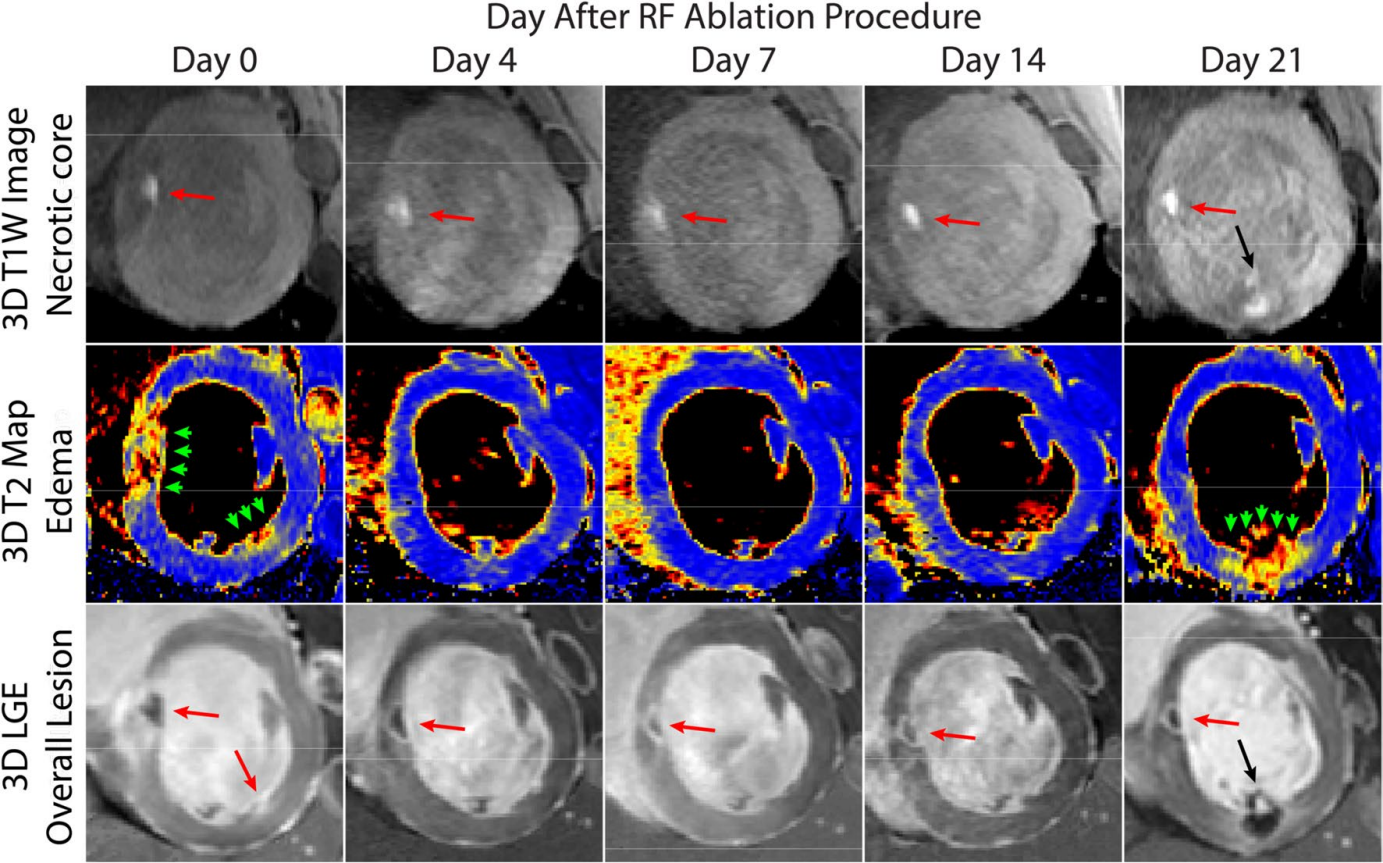
Visualization of radiofrequency ablations in swine. Top row: T1-weighted images identify coagulation necrosis and demonstrate that the lesion core remains relatively constant in size throughout the imaging period which includes encapsulation and scar deposition. One lesion is small, does not generate a necrotic core, and disappears after peripheral edema (green arrows) fades. Middle row: 3D T2-Mapping demonstrates that edema fades quickly before the 4th day. Bottom row: Late gadolinium enhancement (LGE) imaging can easily visualize two lesions (red arrows) applied on Day 0. On Day 21, a new lesion is applied (black arrows) creating new edema and a necrotic core. All animal experiments performed according to institutional guidelines

More recently, T1-weighted MRI using a long inversion time and two-beat triggering (TWILITE) [72] has been demonstrated as a method for lesion visualization in both ventricles and atria (Fig. 7) [67, 72, 73], although this sequence is sensitive to heart rate and arrhythmias which may limit its applicability in patients undergoing arrhythmia ablation. Tissue necrosis produced by heating during RF ablation results in coagulation necrosis with shortened T1 (likely from paramagnetic met-form globin proteins). The area of T1-weighted signal enhancement post-ablation correlates well with necrosis, making the technique suitable for contrast-agent-free inter- and post-procedural assessment of RF ablations feasible. Early results demonstrating its application in pulmonary vein isolation as used in treatment of atrial fibrillation (AF) are encouraging [73].

## What iCMR procedures will we be able to do in the future?

### Endomyocardial biopsy

**Fig. 8:**
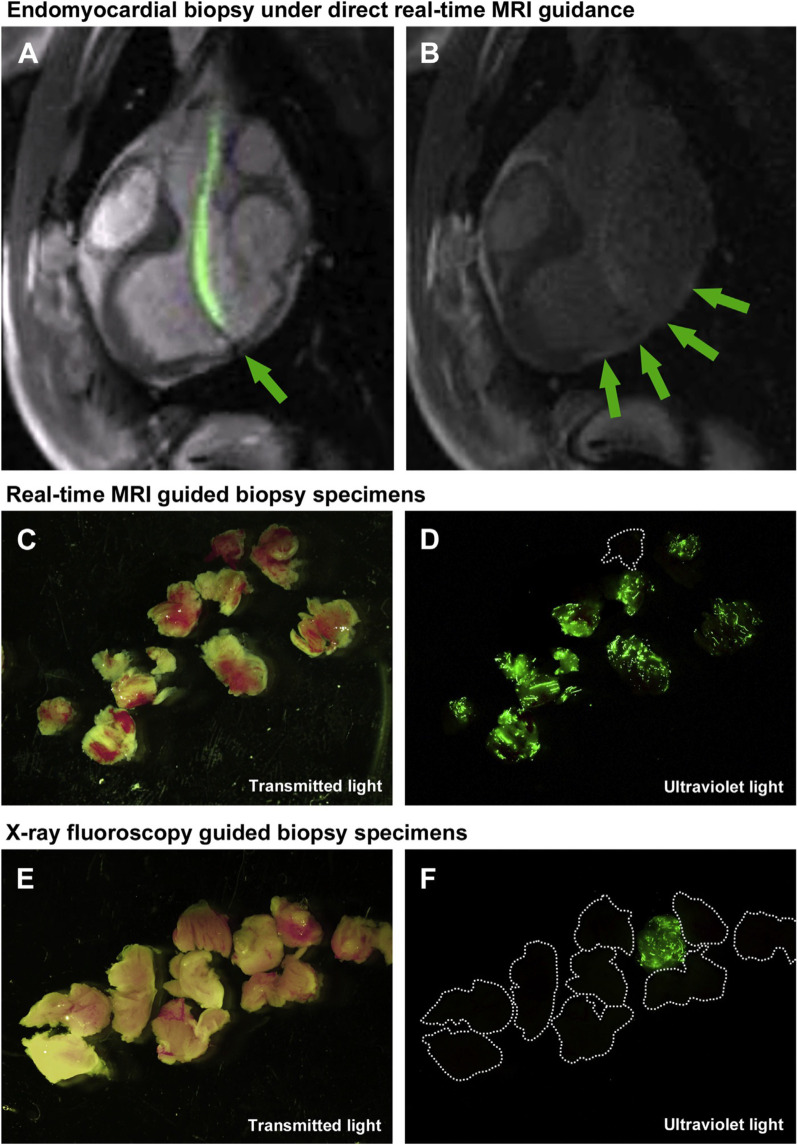
iCMR guided endomyocardial biopsy. **A** Rapid frame-rate real-time MRI-guided navigation of an active visualization iCMR bioptome to target a focal lesion within the left ventricle, labeled with fluorescent microspheres. The jaws appear as a passive artifact (arrow). **B** After systemic gadolinium contrast administration, the lesion is visible using inversion-recovery real-time MRI (arrows). Real-time MRI-guided biopsy specimens viewed under (**C**) transmitted light and (**D**) ultraviolet light demonstrate higher diagnostic yield compared with X-ray fluoroscopy–guided biopsy specimens viewed under (**E**) transmitted light and (**F**) ultraviolet light. Reproduced from Rogers et al. JACC: Basic to Translational Science, 2016. *iCMR* interventional cardiovascular magnetic resonance

Endomyocardial biopsy is typically performed with X-ray fluoroscopy guidance. In essence, this means that biopsy specimens are collected randomly from the endomyocardium in a non-targeted fashion. While this approach is effective in disease processes that affect the myocardium diffusely, the diagnostic yield in patients with patchy myocardial involvement can be low. Furthermore, complications such as tricuspid valve injury leading to regurgitation and right ventricle free wall injury leading to pericardial effusion and tamponade, are not infrequent. Echocardiographic guidance may help to prevent these complications [74], but iCMR guidance for endomyocardial biopsy is especially attractive because of the potential to exploit CMR tissue characterization techniques to identify abnormal tissue to target for biopsy in addition to the ability to visualize and therefore avoid fragile structures such as the tricuspid valve apparatus [75]. Unfortunately, MRI conditional bioptomes are still in development and not yet available for clinical use, but pre-clinical feasibility has been demonstrated using both passive [76] and active [75] bioptome devices (Fig. 8).

### Extra-anatomic bypass

iCMR is particularly appealing for novel procedures that are difficult to perform or envision with X-Ray guidance alone. X-Ray contrast angiography offers excellent imaging of patent blood vessels. Occluded blood vessels are challenging and may require alternate imaging techniques. In contrast, real-time CMR can visualize chronic total occlusion vessels and potentially guide recanalization [77]. Similarly, iCMR may be ideal for transcatheter connection of two different vessels. The enabling feature is that real-time CMR offers continuous imaging in any plane. In the extra-anatomic bypass example, there is continuous imaging of donor and target vessels during all the following key procedural steps: trajectory planning, donor vessel exit, target vessel entry, device delivery system passage, and anastomosis device placement. There is also a large field of view for continuous visualization of critical neighboring structures and early detection of potential complications such as bleeding [78].

One representative example is non-surgical, closed chest, transcatheter superior vena cava (SVC) and pulmonary artery (PA) anastomosis [48]. In this pre-clinical experience, real-time CMR guided all procedural steps for transcatheter cavopulmonary anastomosis (“Glenn” surgery equivalent required for all single ventricle congenital heart disease patients). It was most valuable for two critical steps. An MRI compatible? Needle exited the SVC and was guided along a curvilinear trajectory around the aorta to enter the PA avoiding the right upper pulmonary artery. Second, real-time CMR guided distal anastomosis endograft placement. The endograft must be distal enough to avoid catastrophic miss and proximal enough to allow SVC blood diversion to both right and left pulmonary arteries (“bidirectional” Glenn). This work inspired the first-in-human transcatheter Glenn performed with X-Ray guidance by the same operator group [79]. X-Ray guidance is more challenging than real-time MRI guidance for this procedure because important anatomic structures are radiolucent. Repeated, intermittent contrast injections and marker catheter devices provide a less than ideal substitute for reliable continuous visualization of donor and target vessels and important surrounding structures. Extra-anatomic bypass highlights the appeal of real-time CMR guidance for novel transcatheter procedures.

### Chemoablation

Myocardial ablation performed routinely by cardiac electrophysiologists to alter arrhythmogenic substrate can substantially reduce the burden of or eradicate symptomatic arrhythmia. Ventricular tachycardia (VT) is frequently a sinister, malignant diagnosis with heterogeneous melee of etiologies. Common to all is the requirement for differential rates of myocardial conduction. The aim of ablative therapy is to create a more confluent non-conductive lesion, eradicating areas of slow or rapid conduction within pathological myocardium. Successful radiofrequency ablation (RFA) therefore requires localization through complex mapping, dexterity both of the ablation equipment and operator to reach the target and consistent catheter contact to delivery sufficient energy to induce coagulation necrosis and destroy the target.

MRI guided chemoablation holds promise as an alternative to RFA and has been performed successfully in the pre-clinical setting [80, 81]. Attractive features of MRI guided chemoablation include direct substrate visualization and active needle tracking which permit accurate lesion targeting and agent delivery. Using these features, proof of concept has been demonstrated in an animal model of ischemic cardiomyopathy when an isthmus of normally conducting myocardium was ablated between two areas of fibrosis [80]. The chemoablation injectate (50% acetic acid) was doped with gadolinium and injected under real time inversion-recovery MRI allowing visualization and tracking of lesion development. Moreover, hyperenhancement of the acute lesion in this setting accurately predicted chronic lesion location and volume [80, 81]. This has the potential to increase procedural efficacy, offsetting some of the variability encountered with in vivo lesion creation. Some attractive targets for this therapy that are classically troublesome to ablate with RF techniques include intra-septal VT, VT that arises from the cardiac summit, epicardial VT and VT arising from complex, ischemic scar.

### Ventricular tachycardia ablation

iCMR guided EP has greatest promise for treating complex arrhythmias like ventricular tachycardia (VT) where (1) CMR can detect the arrhythmogenic myocardial scar “substrate” and (2) currently poor treatment outcomes are thought to be related to incomplete ablation of arrhythmogenic tissue. Because iCMR is inherently registered to CMR substrate and ablation lesion images, catheters can be accurately guided to areas of substrate and incomplete ablation and additional ablation performed. Lesion imaging methods that utilize native tissue contrast also permit repeated lesion assessment until complete ablation is confirmed [72, 82, 83]. This paradigm is attractive for guiding VT ablation because CMR is the gold-standard for transmural delineation of scar substrate, whereas conventional mapping is limited at detecting scar substrate deeper within the myocardium [84]. Post-ablation lesion assessment also appears useful for guiding VT ablation, since ablating arrhythmogenic tissue deeper within the myocardium is challenging, particularly in regions of scar [67]. CMR detection of incomplete ablation at deeper myocardial sites could prompt use of alternative ablation techniques [80]. Integration of myocardial scar CMR and ablation lesion CMR is also possible with conventional mapping guided procedures [84–86]. However, repeated transfer of patients between the EP lab and MRI scanner is inefficient and has limited use of post-ablation lesion CMR to guide additional ablation. In addition, the process of registering conventional mapping systems to CMR is error prone which limits accuracy of catheter guidance.

Most procedural elements needed for iCMR guided VT ablation have been demonstrated in the preclinical setting. The first report of iCMR guided EP ablation involved right ventricle electrogram measurement and ablation [87]. Since then more clinical approaches to VT mapping and ablation have been implemented including iCMR guided left ventricular access by retrograde aortic, transseptal, and epicardial approaches [88–90], iCMR guided endocardial and epicardial voltage and activation mapping [70, 91, 92], and iCMR guided left ventricular endocardial and epicardial ablation [70, 93]. Low amplitude electrograms features corresponding to slow electrical propagation within scar help direct conventional ablation and also appear detectable inside the MR scanner [92]. Induction of VT is an initial step in grossly localizing arrhythmia by ECG appearance and is a final step in evaluating potential ablation targets. The ability to defibrillate/cardiovert hemodynamically unstable ventricular arrhythmia is often required. Safe defibrillation inside MRI scanner has been demonstrated in animal models [34, 35].

The availability of a commercial MRI conditional EP system has permitted transition from pre-clinical to clinical iCMR guided ablation procedures [15, 16, 18]. The first trial of iCMR VT ablation in patients with ischemic cardiomyopathy is due to begin enrollment in 2023 (NCT05543798). The 12-lead ECG is typically used for initial anatomic localization of VT and for “pace-mapping” localization of hemodynamically unstable VT [94]. Distortion of the QRS complex by MHD is likely to require modified localization methods, improved MHD correction techniques [24], or operation under low-field conditions. MRI conditional multi-electrode catheters with smaller electrodes [54] will also be beneficial for more rapidly detecting the localized low amplitude signals that can identify ideal targets for ablation. Most patients who present for VT ablation also have implantable cardioverter defibrillators (ICDs). Older ICDs were typically MRI Unsafe, however newer ICD technology, standardized monitoring and device programming protocols have been applied to perform MRI in more than 3000 patients without significant adverse effects [95, 96]. The effect of performing long iCMR procedures in patients with ICDs requires further study. Independent of safety considerations, ICDs create strong magnetic field distortions in the MRI scanner, which can lead to significant image artifacts. Methods for mitigating image artifacts for inversion-recovery imaging of myocardial scar have been described [97, 98]. However, CMR image quality with balanced-SSFP imaging can still be compromised in ICD patients [99] and will require adapted imaging protocols.

## Conclusions

Until recently, only higher field strength MRI scanners had hardware capable of imaging the beating heart. This has hampered the development and clinical roll out of iCMR procedures because commercially available metallic guidewires, catheters and devices had to be individually redesigned to make them conspicuous and free from heating. Now, low-SAR pulse sequences, dedicated catheter and device engineering, and availability of low field MRI scanners with advanced hardware provides an opportunity for a new generation of iCMR applications.

## Data Availability

Not applicable for this review manuscript.

## Abbreviations

3D: Three-dimensional
AF: Atrial fibrillation
bSSFP: Balanced steady state free precession
CMR: Cardiovascular magnetic resonance
ICD: Implantable cardioverter defibrillator
iCMR: Interventional cardiovascular magnetic resonance
EP: Electrophysiology
ECG: Electrocardiogram
GRAPPA: Generalized autoencoding partially parallel acquisition
HVAC: Heating, ventilation and air conditioning
LGE: Late gadolinium enhancement
MHD: Magnetohydrodynamic
MRI: Magnetic resonance imaging
NHLBI: National Heart Lung and Blood Institute
PA: Pulmonary artery
RF: Radiofrequency
RFA: Radiofrequency ablation
SENSE: Sensitivity encoding
SAR: Specific absorption rate
SNR: Signal to noise ratio
SVC: Superior vena cava
VT: Ventricular tachycardia
